# How Repeatable Is the Ergogenic Effect of Caffeine? Limited Reproducibility of Acute Caffeine (3 mg.kg^−1^) Ingestion on Muscular Strength, Power, and Muscular Endurance

**DOI:** 10.3390/nu14204416

**Published:** 2022-10-21

**Authors:** Ryan A. Tamilio, Neil D. Clarke, Michael J. Duncan, Rhys O. Morris, Jason Tallis

**Affiliations:** Centre for Applied Biological and Exercise Science, Alison Gingell Building, Coventry University, Priory Street, Coventry CV1 5FB, UK

**Keywords:** ergogenic-aids, repeatability, strength, power, resistance exercise

## Abstract

This study aimed to determine the effect of 3 mg.kg^−1^ acute caffeine ingestion on muscular strength, power and strength endurance and the repeatability of potential ergogenic effects across multiple trials. Twenty-two university standard male rugby union players (20 ± 2 years) completed the study. Using a double-blind, randomized, and counterbalanced within-subject experimental design. Participants completed six experimental trials (three caffeine and three placebo) where force time characteristic of the Isometric Mid-Thigh Pull (IMTP), Countermovement Jump (CMJ) and Drop Jumps (DJ) were assessed followed by assessments of Chest Press (CP), Shoulder Press (SP), Squats (SQ), and Deadlifts (DL) Repetitions Until Failure (RTF at 70% 1 RM). ANOVA indicated that caffeine improved both the CMJ and DJ (*p* < 0.044) and increased RTF in all RTF assessments (*p* < 0.002). When individual caffeine trials were compared to corresponding placebo trials, effect sizes ranged from trivial-large favoring caffeine irrespective of a main effect of treatment being identified in the ANOVA. These results demonstrate for the first time that the performance enhancing effects of caffeine may not be repeatable between days, where our data uniquely indicates that this is in part attributable to between sessions variation in caffeine’s ergogenic potential.

## 1. Introduction

The ergogenic potential of caffeine to evoke improved physical and cognitive performance has been firmly established [1,2,3]. Numerous meta-analyses have demonstrated small but significant improvements in endurance [4,5], muscular function [5,6,7] and sport-specific skills [8,9] following acute caffeine ingestion. As such, caffeine has become a popular nutritional supplement to improve sports performance [9,10,11].

Despite well-established and overwhelmingly reported beneficial effects, there remains a wealth of evidence indicating limited or no acute caffeine effects on measures of muscular strength and power [1,5,8,12] and are considered more equivocal compared to other physical performance measures. Equivocal results have been attributed to methodological discrepancies such as the muscle group assessed, methods of quantifying strength/power, participants caffeine consumption habits, training status, the dosage of caffeine administrated, and genotype variation [2,6,13,14,15]. Consequently, researchers continue to investigate the ergogenic effects of caffeine to optimize its performance-enhancing potential.

Where caffeine has been shown to elicit beneficial effects on muscular strength and power performance, effects are generally small in magnitude [2,5,6,7] and conclusions drawn from studies with low sample sizes. Although studies in this area generally employ established methods that are validated and reliable, many studies fail to consider the test–retest reliability of the assessment methods in the specific population examined [16,17,18,19]. Such methodological issues may impact the ability to accurately assess changes following acute caffeine consumption due to the variation in the assessment measure, which may be further compounded by daily variation in an individual’s caffeine response. It is reasonable to believe that an individual’s response to caffeine may differ depending on mood, motivation, and level of fatigue (inclusive of sleep). Mood [20], motivation [21] and fatigue [22,23,24] independently influence exercise performance and acute caffeine ingestion has been shown to increase mood [25,26], increase motivation for exercise [27,28] and reduce feelings of fatigue [29], which in part accounts its performance enhancing effect.

Almost exclusively, previous work examining the acute effects of caffeine has compared performance in a single treatment trial to that of a controlled trial (i.e., placebo/non-caffeine trial) [2,30,31], where error introduced by variation in repeatability in the assessed outcomes and variation in response to caffeine ingestion may result in misleading conclusions when attempting to measure small effects. The present study is the first to investigate the acute effects of the same caffeine dose over multiple trials, and as such, will uniquely identify potential between daily variation in caffeine erogeneity and its influences on drawing meaningful conclusion regarding its performance enhancing effect. 

Given that the effect of caffeine may be influenced by assessment mode, contraction modality and maybe muscle group-specific [32,33,34], the present study will examine the repeatability of caffeine’s ergogenic effects on the force-time characteristics of the isometric-mid thigh pull (IMTP), countermovement jump (CMJ), and drop jump (DJ). Strength endurance will be assessed using repetitions until failure (RTF) assessment of upper and lower body muscle groups. Benefits of caffeine have been previously demonstrated individually for these exercises [5,6,7,35,36]. This study therefore provides novel insight with respect to the effect of acute caffeine ingestion on measures of muscular strength and power and more specifically aimed to (i) determine the effect of 3 mg.kg^−1^ acute caffeine ingestion on muscular strength, power, and strength endurance, (ii) to determine if 3 mg.kg^−1^ caffeine on muscle functions is repeatable across multiple trials.

## 2. Materials and Methods

### 2.1. Participants

Following ethics approval from the host institute [P94037] and written informed consent, 40 apparently healthy participants from the Coventry University Men’s Rugby Union team participated in the study. Participants completed a health screen questionnaire at each visit and were excluded if suffering from a musculoskeletal injury that prevented safe completion of the exercise trials, consumed psychoactive medication, or had any other underlying contradictions to exercise. From the original sample, 18 dropped out due to injury (*n* = 8) (unrelated to the experimental protocol), illness (*n* = 3), or for reasons unstated (*n* = 7) leaving a sample of 22 (Mean ± SD Age (years) 20 ± 2; Height (cm) 181 ± 7; Body Mass (kg) 91 ± 23). Participants completed a caffeine consumption questionnaire [37] to provide an estimate of typical daily consumption habits. On average, participants consumed 118 ± 88 mg per day (moderate range: 104–183) with 8 participants reporting no caffeine use. 

### 2.2. Experimental Design

**Figure 1:**
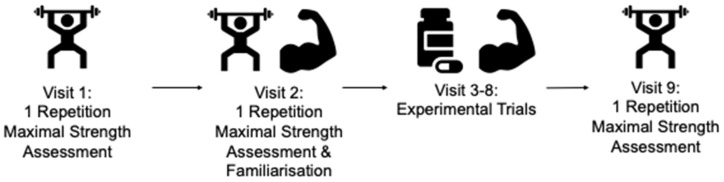
Schematic of Experiential design.

Participants were asked to attend the research facility at the host institute on nine separate occasions (Figure 1). Initially, an assessment of one repetition maximal strength (1 RM) was conducted followed by reassessment of 1 RM and familiarisation to the experimental procedures. Following this, the repeatability of acute caffeine effects on muscular strength, power and muscular endurance was assessed across 6 experimental trials (3 caffeine & 3 placebo) using a double-blind, randomized, and counterbalanced within-subject experimental design. Upon completion of the experimental trials, a subsequent 1 RM re-test was completed to identify potential training effect induced by multiple trials. 

Participants were asked to abstain from caffeine and intense physical exercise for 12 and 48 h, respectively, in accordance with previous work [26]. When prescribed, participants consumed either caffeine (3 mg.kg^−1^; Myprotein, Manchester, UK) or a placebo (3 mg.kg^−1^; maltodextrin; Myprotein, UK). In each case, the required dose was determined for each individual and transferred to a transparent gelatin capsule (BulkTM, Colchester, UK). 3 mg.kg^−1^ is regularly cited as a minimal dose needed to evoke a physical performance enhancement [4,38] and previously used when assessing caffeine’s effect on muscular strength and power [5,6,7].

### 2.3. RM

Participants completed a 1 RM for Chest Press (CP), Shoulder Press (SP), Squats (SQ) and Deadlifts (DL), assessments that have been used previously to evaluate acute effects of caffeine on muscle function [39]. Participants conducted a warmup including static and dynamic stretches with inclusive 8–10 unweighted warm-up repetitions using a 20 kg Eleiko bar (Pullum Power Sports, Luton, UK). All participants received a demonstration of the correct lifting techniques [40]. All participants had previous experience with all exercises. A trained researcher/spotter was present to ensure safety and proper range of motion. Exercises were completed in the following order CP, DL, SP, then SQ. Prior to 1 RM attempts participants estimated 50% and completed 3–5 repetitions, progressing to 70% for 1–3 repetitions, and onto 90% for 1 repetition. 1 RM was determined by progressively increasing the weight (minimum of 2.5 kg incline) [18] lifted until the participant failed to lift the set of weights through a full range of motion [41] and/or the technique did not correspond to guidelines for the execution of the exercises as outlined by Baechle and Earle [40]. Exercises was altered between upper and lower body to alleviate fatigue with a minimum of 1-min rest between attempts and 5 min between lifts [42].

### 2.4. Familiarisation

Initially, participants removed shoes and heavy clothing, and measures of height (cm) and body mass (kg) were taken using a stadiometer (SECA 213, Hamburg, Germany) and electronic weighing scales (SECA 803, Hamburg, Germany) respectively. 1 RM was then reassessed and then participants familiarised to the CMJ, DJ, IMTP, and RTF testing procedures.

### 2.5. Countermovement Jump

CMJ’s were performed on two PASCO force plates (PASCO, Scientific, Roseville, CA, USA) sampling at 1000 Hz. Participants were instructed to have their arms akimbo (fixed on the hips) and to remain this way during the movement. Participants started on the force plates in a standing upright position and executed the movement following at least 1 s of quiet standing [43]. Participants were instructed to jump as high and as fast as possible. All participants completed three successful jumps with a minimum of 30 s rest between attempts. Raw vertical force time data was collected and exported from PASCO Capstone software (Capstone software version 1.13.4). Data was mathematically integrated as per the procedure outlined by Chavda et al. [44]. Jump height (JH) (m) was determined from take-off velocity (TOV) (JH = TOV^2^/2 g) [44,45,46]. Where TOV is the vertical velocity (V) at take-off (TO) and g is the gravitational acceleration (9.81 m.sec^−2^). Using the attempt that elicited the greatest JH, reactive strength index modified (RSI mod) (JH /Time to TO), Peak Force (PF) (greatest Vertical Force (Fz) prior to TO), Peak Power (PP), Concentric Impulse (Ns) and Eccentric Impulse (Ns) were determined as be the procedures outlined by Chavda et al. [44] with PF and PP being presented relative to body mass.

### 2.6. Drop Jump 

DJs were performed on two bilateral PASCO force plates sampling at 1000 Hz. Participants started standing upright on a box 45 cm off the ground compensating for the height of the force plate (5 cm) resulting in a 40 cm drop height. Participants with arms akimbo remained in this position through the entire jump. Participants were instructed to jump as high and as fast as possible and executed the movement by stepping off the box with their dominant leg leading and upon landing, instantaneously performed a maximal vertical jump. A successful DJ was determined by the arms being akimbo, stepping off the box leading with the dominant leg, and upon landing on the two force plates simultaneously. Jumps that deviated from this were repeated. Participants completed 3 successful jumps, separated by a minimum of 30 s rest. Backwards integration (BI) of raw unfiltered vertical force time data, as per the method outline by Wade et al. [47], was used to determine vertical velocity of the centre of mass. From which JH was determined via TOV. Using the attempt that elicited the highest JH, PF, PP, and RSI were determined as per published methods [47,48]. 

### 2.7. Isometric Mid-Thigh Pull

IMTPs were performed on floor integrated triaxle force plates (AMTI, ACP-A, Waterton, MA, USA) sampling at 1000 Hz. IMTP was assessed using a custom-built steel rack fixed to the ground. Participants were asked to stand with a knee angle of 135–145° and a hip angle of 140–150° [49,50] with their shoulders placed above the bar [51]. Joint angles were checked using a goniometer. Lifting straps were used to reduce the loss of grip [52]. Following the collection of a stable force trace, participants pulled upwards in a maximal fashion [52] for a duration of ~5 s then rested for 2 min in-between repetitions [49]. Participants received loud verbal encouragement. Participants completed three successful attempts and were reassessed if there was evidence of a countermovement [49,53]. Raw unfiltered vertical force data were extracted for analysis using AMTINetForce software (AMTI Bio Analysis Software, NetForce, 2.4.0). Analysis was performed on the attempt that elicited the highest peak force. Initiation of movement was determined by using a 5 SD threshold calculated from 1 s of quiet standing force gathered prior to each pull attempt [50,53]. PF (N.Kg), Time to Peak Force (TTPF) (second), PF (N) at 100 m/s (F100) and 300 (F300) m/s were calculated. These variables were chosen to provide theoretical representation of an individual’s ability to produce maximal force and perform tasks associated with slow and fast stretch-shortening cycles [54].

### 2.8. Resistance Exercise 

Following the warm-up outlined in the 1 RM protocol, participants completed an RTF assessment of CP, SP, SQ, and DL. Participants completed two sets of RTFs at 70% 1 RM as per previous work [25,26,55]. Previous work using RTF protocol commonly use loads of 60–80% of 1 RM [56,57] given such loads have suggested to be effective for improving muscular strength and endurance [58]. A trained researcher/spotter was present during all resistance exercises ensuring proper range of motion and any lift that deviated from guideline outlined by Baechle and Earle [40] was not counted. Exercises were completed in the following order: CP, DL, SP then SQ altering from upper to lower body. There was a minimum of 2-min rest in-between exercises and a 10-min rest between circuits. Total repetitions were recorded on completion of each exercise and RPE assessed using the Borg scale [59]. 

### 2.9. Experimental Trials

**Figure 2:**
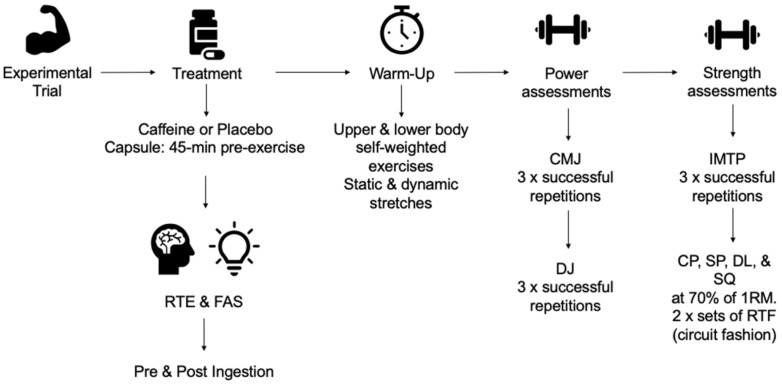
Schematic of Experimental Trial.

Prior to completing the exercises outlined above, participants ingested either a caffeine or a placebo treatment 45-min prior to experimental trials [2] as maximal blood plasma concentration of caffeine occurs 40–60 min post-consumption [38,60]. Readiness to Invest Effort (RTE) Physical and Mental [26] and Felt arousal scale (FAS) [61] were completed pre-and 45 min post ingestion (Figure 2) to assess state motivation and arousal levels prior to exercise.

### 2.10. Data Analysis 

Data analysis was performed using Statistical Package for the Social Sciences (IBM SPSS Statistics Version 25) and Excel (Microsoft Windows Version 16.41 2020). In order to identify a potential training effect from continuous bouts of resistance exercise pre-and-post-experimental 1-RM performance was evaluated using paired t-test. Test-re-test reliability of each variable was measured between all placebo trials (T1, T2, and T3) and caffeine trials (T1, T2, and T3). For each comparison, Interclass correlation (ICC) was performed, and 95% CI determined, with reliability being classed as: Poor (<0.50), Moderate (0.50–0.74), Good (0.75–0.89), and Excellent (0.90<) [62]. Coefficient of Variation (CV) was determined using the equation SD ÷ the Mean x 100 [63]. Bland–Altman’s assessment and 95% limits of agreement was also determined [64,65]. In order to determine the effects of caffeine, RTE and FAS were assessed using a 3-factor repeated-measures ANOVA with Treatment (caffeine or placebo), Trial (Trial 1, 2 or 3), and Time (pre-and post-ingestion) as factors. Acute effects of caffeine on CMJ, DJ, and IMTP were analysed using a 2-factor repeated measure ANOVA with Treatment (caffeine or placebo) and Trial (Trial 1, 2, or 3) as factors. RTF and RPE were analysed using a 3-factor repeated-measures ANOVA with Treatment (caffeine or placebo), Trial (Trial 1, 2 or 3), and Set (Set1 or Set2) as factors. For ANOVA, Greenhouse-Geisser adjustment was interpreted when Sphericity was violated, and relevant significant main effects and interactions were explored via Bonferroni adjusted pairwise comparisons. For ANOVA, Partial eta squared (η^2^) was reported with as a measures of effect size [66] and was categorised as small (0.01), medium (0.06), and large (0.14) [67]. Effect Size for pairwise comparison were explored by determining Cohen’s d corrected for bias using Hedge’s g [68]. Hedge’s g effect size (ES) was interpreted as trivial <0.20, small 0.20–0.49, medium 0.50–0.79, and large >0.80 [69]. Data were presented as Mean ± SD with statistical significance set at a level of *p* < 0.05.

## 3. Results

### 3.1. Effect of Experimental Trials on Maximal Strength

**Figure 3:**
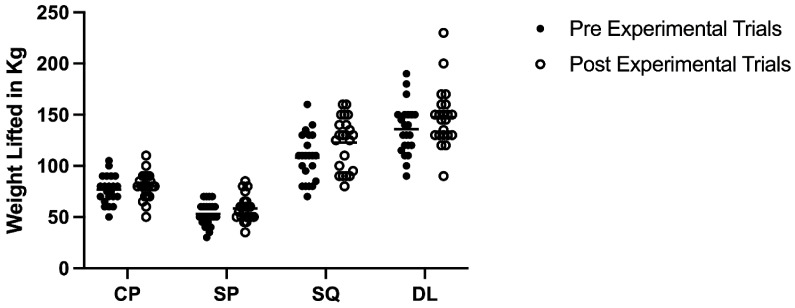
Pre and Post Experimental Trials 1 RM.

Upon completion of the study 1 RM performance did not statistically differ to that prior to completion of the experimental trials in any of the assessed lifts (Figure 3. CP: *p* = 0.437 (ES = 0.22), SP: *p* = 0.149 (ES = 0.39), SQ: *p* = 0.051 (ES = 0.61), DL: *p* = 0.162 (ES= 0.44).

### 3.2. Effect of Caffeine on Arousal and Motivation

Test–retest reliability of the perceptual measures ranged from poor to moderate (Supplementary Table S1: ICC < 0.734, CI = −0.448−0.880) across both treatments. The reported range for CV across placebo trials 19.6–44.2% whereas caffeine trials ranged from 12–34.3%. Bland–Altman’s analysis indicated that both placebo and caffeine trials Lo-Up LOA had high and moderate variation across the measures (Supplementary Table S1).

For RTE Physical, RTE Mental, and FAS there were no significant Treatment*Trial*Time (Table 1: *p* > 0.190 η_p_^2^ < 0.076) or Trial*Time (Table 1: *p* > 0.228 η_p_^2^ < 0.068) interactions. For RTE Mental and FAS there were no Treatment*Trial interactions (Table 1: *p* > 0.068 η_p_^2^ < 0.239). However, there was a Treatment*Trial (Table 1: *p* = 0.019 η_p_^2^ = 0.171) interaction for RTE Physical with pairwise comparison indicating higher RTE Physical in the caffeine trials (Table 1: *p* < 0.013 d > 0.17). Furthermore, RTE Physical, RTE Mental, and FAS there was an interaction between Treatment*Time (Table 1: *p* < 0.050 η_p_^2^ > 0.170). Pairwise comparison for RTE Mental revealed an increase pre-and post-ingestion in the caffeine trial (Table 1: *p* < 0.033 d > 0.26) but RTE Physical and FAS was only higher with caffeine post-ingestion (Table 1: *p* < 0.006 d > 0.24). For RTE mental and FAS there was no main effect of Trial (Table 1: *p* > 0.064 η_p_^2^ < 0.123).

### 3.3. Effect of Caffeine on Countermovement Jump Performance

Test re-test reliability of CMJ measures ranged from good to excellent across both treatments (Supplementary Table S2: ICC > 0.901, CI = 0.485–0.972) apart from RSI (mod), where the ICC was moderate (Supplementary Table S2: ICC < 0.716, CI = 0.355–0.855) for the placebo trials. For all variables the range of CV for placebo trials were 5–18.1% whereas the caffeine trials CV ranged from 5.3–16.4%. Bland–Altman’s analysis indicated consistency in the level of agreement across measures between caffeine and placebo (Supplementary Table S2).

In all cases there was no significant interactions between Treatment*Trial (Table 2: *p* < 0.873 η_p_^2^ < 0.096) or no main effect of Trials (Table 2: *p* < 0.923 η_p_^2^ < 0.100). For eccentric impulse (Ns) there was no effect of treatment (Table 2: *p* = 0.771 η_p_^2^ = 0.004). However, JH (m), RSI (mod), PF (N.kg), PP (W.kg), and concentric impulse (Ns) were all higher following caffeine treatment (Table 2: *p* < 0.044 η_p_^2^ > 0.179).

### 3.4. Effect of Caffeine on Drop Jump Performance 

Test re-test reliability of DJ measures ranged from poor to moderate across both treatments (Supplementary Table S3: ICC < 0.665, CI = −0.257–0.665) apart from RSI where the ICC was good and excellent reliability (Supplementary Table S3: ICC > 0.857, CI = −0.687–0.982). For all variables, the CV for the placebo trials ranged from 7–26% whereas caffeine trials ranged from 7.8–25.1%. Bland–Altman analysis indicated consistency in the level of agreement across measures between caffeine and placebo (Supplementary Table S3).

In all cases there was no Treatment*Trial interactions (Table 3: *p* < 0.924 η_p_^2^ < 0.027) and no main effects of Trial (Table 3: *p* < 0.876 η_p_^2^ < 0.047). For, RSI and PF there was no effect of Treatment (Table 3: *p* < 0.633 η_p_^2^ < 0.094) however, JH and PP were higher in the caffeine (Table 3: *p* < 0.042 η_p_^2^ > 0.183). 

### 3.5. Effects of Caffeine on Isometric Mid-Thigh Pull

Test–retest reliability of IMTP measure of TTPF and F100, during both treatments was varied (Supplementary Table S4: ICC < 0.800, CI = −0.339−0.912) apart from PF during caffeine trials which was good/excellent (Supplementary Table S4: ICC < 0.923, CI = 0.648–0.967) and F300 during placebo trials was moderate/good (Supplementary Table S4: ICC < 0.800, CI = 0.404–0.912). The CV for placebo trials ranged from 8–39.1% whereas CV in the caffeine trials ranged from 4.3–39.8%. Bland–Altman’s analysis indicated consistency in the level of agreement across measures between caffeine and placebo trials (Supplementary Table S4).

There was no Treatment*Trial (Table 4: *p* < 0.970 η_p_^2^ < 0.067) interaction, main effects of Trials (Table 4: *p* < 0.732 η_p_^2^ < 0.052) or Treatment (Table 4: *p* < 0.705 η_p_^2^ < 0.125) for any of the measured variable.

### 3.6. Effect of Caffeine on Repetition until Failure

Test–retest reliability of Set1 and Set2 CP, SP, SQ, and DL RTF during both treatments was varied (Supplementary Table S5: ICC < 0.746, CI = −0.178–0.899). The CV for placebo trials ranged from 11.2–22.9% whereas caffeine trials ranged from 8.7–23.7% Bland–Altman analysis for all variables during both treatments suggest a relatively high variability with LOA and mean bias (Supplementary Table S5).

For RTF CP, SP, DL, SQ there were no Treatment*Trial*Set (Table 5: *p*< 0.888 η_p_^2^ < 0.061), Treatment*Trial (Table 5: *p* < 0.846 η_p_^2^ < 0.065), Trial*Set (Table 5: *p* < 0.956 η_p_^2^ < 0.006), and Treatment*Set (Table 5: *p* < 0.888, η_p_^2^ < 0.017) interactions. However, for SQ RTF there was a Treatment*set (Table 5: *p* = 0.048 η_p_^2^ = 0.174) interaction with pairwise comparison indicating caffeine trials had higher RTF (Table 5: *p* = 0.001 *d* < 0.28) as well higher performance in Set1 compared to Set2 in both cases (Table 5: *p* = 0.001 *d* < 0.*25).* In all cases there was no main effect of Trial (Table 5: *p* < 0.975 η_p_^2^ < 0.119) apart from SP RTF (Table 5: *p* = 0.047 η_p_^2^ = 0.136) with Trial 1 having a greater RTF. In all other cases RTF were greater in Set1 compared to Set2 (Table 5: *p* = 0.001 η_p_^2^ > 0.710) and there was a main effect of Treatment (Table 5: *p* < 0.002 η_p_^2^ > 0.365) where performance in the caffeine trial was improved.

### 3.7. Effect of Caffeine on Rate of Perceived Exertion

Test–retest reliability of Set1 and Set2 RPE was poor or moderate (Supplementary Table S6: ICC < 0.728, CI = −0.502–0.878). The CV for placebo trials ranged from 2.1–9.8% whereas caffeine trials ranged from 2.2–6.1%. Bland–Altman analysis for all Set1 and Set2 indicated consistency in the level of agreement across measures between caffeine and placebo trials (Supplementary Table S6).

In all cases there was no Treatment*Trial*Set (Table 6: *p* < 0.896 η_p_^2^ = 0.055), Treatment*Set (Table 6: *p* > 0.107 η_p_^2^ < 0.129), or Trial*Set (Table 6: *p* < 0.768 η_p_^2^ < 0.102) and Treatment*Trial interactions (Table 6: *p* < 0.514 η_p_^2^ < 0.075) apart from SQ which had a significant Treatment*Trial interaction (Table 6: *p* = 0.019 η_p_^2^ = 0.173). Pairwise comparison indicated trial 2 Set1 RPE was significantly higher in the Placebo treatment than the Caffeine treatment (Table 6: *p* = 0.032 d = 0.098). All exercises had no main effect of Treatment (Table 6: *p* < 0.402 η_p_^2^ < 0.115). CP, SP, and DL had no significant main effect between Trials (Table 6: *p* < 0.720 η_p_^2^ < 0.183). In all cases, RPE was higher following completion of the second set (Table 6: *p* = 0.000 η_p_^2^ < 0.842).

## 4. Discussion

The present study is unique in examining the test–retest reliability and repeatability of 3 mg.kg^−1^ acute caffeine consumption on measures of muscular function, offering important insight into performance-enhancing effects of caffeine When examined collectively across three repeated trails, caffeine enhanced specific measures of muscular strength, power, and strength endurance. However, when individual caffeine trials were compared to placebo, effects varied from trivial to large, irrespective of the main effect of treatment being identified via ANOVA. These finding therefore indicate that in some cases caffeine may elicit performance enhancing effects in some trials and not in others. Our data suggest that this varied response may be caused by a lack of reliability in selected outcome measures and/or between sessions variation in caffeine’s effect. These results may help to contextualise ambiguity in previous results examining the acute effects of caffeine on muscular strength and power, but importantly indicate a need for future work to assess the ergogenic potential of caffeine across multiple trials to prevent misleading conclusions and provide a more accurate understanding of caffeine’s effect.

### 4.1. Acute Effect of Caffeine on Muscular Strength and Power 

The present study identified some overall treatment effects during measures of muscular function (e.g., CMJ JH, PF, and DJ JH, and PP in addition to RTF for all exercises were improved in the caffeine trial), and in some cases, there was no main effect of treatment but caffeine effects prevalent when single caffeine trials were compared to placebo (e.g., IMTP PF, TTPF, and F100). Although, it must be noted these effects were predominantly small (*d* > 0.20–0.49). Recent meta-analyses have identified the benefits of caffeine on measures of muscular strength and power, inclusive of CMJ, DJ, and RTF [5,6]. Effects of caffeine on IMTP performance have been considered far less frequently, however, the lack of effect following caffeine ingestion aligns with previous work by Burke et al. [70] who demonstrated 6 mg.kg^−1^ caffeine had no effect on IMTP performance of non-specifically resistance trained female but contradicts work by Harty et al. [71] who showed increased performance in a male population when supplemented with the same dose. When examined at the single trial level, caffeine elicited a small increase in performance on some occasions, however, this should be considered in the context of higher between session errors.

Performance enhancing effect of caffeine has been shown to be muscle group specific [1,5,72], with conflicting evidence proposing greater effect in the lower body muscle function compared to upper [1] or the reverse [5]. The present data fail to support differential effects for upper body and lower body musculature, with caffeine induced benefits prevalent in both upper (e.g., CP and SP, RTF) and lower limb measures (e.g., DJ JH and PP and CMJ RSI (mod), PF, PP, and concentric impulse) of muscle function. Our results do however support the idea of a contractile mode specific effect [33], with benefits evident for outcomes derived from dynamic muscle function assessments, but not in isometric tests (i.e., IMTP).

Though the purpose of this study was not to determine the mechanisms underpinning acute caffeine, some inferences can be made from examination of the perceptual measures. Benefits attributed to caffeine’s potential analgesic effects [4,73,74] can be ruled out, given that in most cases, caffeine induced benefits in RTF were apparent with limited changes in RPE. Despite evidence supporting caffeine suppressed perception of effort as a mechanism for improved performance [18,55,75] the current findings support previous work that contradicted this suggestion [4,73,74]. However, albeit it indirectly, data in the present study supports the idea that caffeine may impart elicit performance enhancing effects on dynamic muscular function via increasing excitatory neurotransmitters, with evidence of increased RTE and FAS post caffeine ingestion. Such effects are not always prevalent in previous work [73,76] which maybe a result of mixed between sessions reliability of perceptual measures as indicated in our data. However, results of the present study support the general idea that caffeine influences mood and motivation [26,77,78].

### 4.2. Reliability and Repeatability of Acute Caffeine Ingestion on Muscular Strength and Power

This present work is the first to examine the repeatability of caffeine’s acute effects on muscular function and it is evident from the data presented, that the beneficial effects of acute caffeine consumption may not be apparent when specific caffeine and placebo trials are compared. The present work therefore indicates that acute caffeine may not be repeatable, suggesting an overhaul in the experimental design typically utilised is assess caffeine ergogenicity may be needed to be able to accurately assess caffeine effect on performance, particularly where effects are likely to be small.

Despite meta-analyses identifying positive effects of acute caffeine consumption on muscular function [2], there are still a number of studies that fail to demonstrate an effect [16,77,79]. Ambiguity in findings has commonly been attributed to, genetic differences in caffeine metabolism [80], differences in dose and method of administration [7,15], performance level of the participants [5], and differences in the assessment methods [2,5,6]. Whilst these factors will likely influence the effect, our results infer that a primary contributor to conflicting results seen in previous work is a lack of between session reliability in the outcome measures utilised and between session disparities in caffeine’s effect, despite controlled conditions. Previous work almost exclusively assesses the effect of acute caffeine ingestion on performance using a single trial [2], which based on the variation in response seen in the present study, may result in misleading conclusions.

In the present study, no main effect of treatment was reported for some outcome measures (i.e., IMTP), however, there were trivial to large effects when specific caffeine trials were compared to placebo (e.g., IMTP PF, TTPF, and F100). Conversely, in other cases, trivial to large effects were prevalent for specific comparisons in the presence of a main effect of treatment (for example CMJ JH, PF and DJ JH, PP in addition to RTF of all exercises). Given that acute caffeine ingestion is typically associated with small significant increase in performance [2], it is evident from the data presented in the current study, that in some cases the varied effect can likely be attributed to an inability to accurately detect a difference (or lack of) in outcome measures that have lower or varied between session reliability. Whilst many of the assessments used in the present study are commonplace in previous work [1,2,5,6,7,35] and in most cases the between session reliability has been established in independent studies [19,81,82,83,84,85] many studies examining the acute effect of caffeine on muscle function fail to assess reliability of the chosen outcome measures in the specific population examined or justify between session reliability using previous work where the population may differ to that recruited.

Poor between session reliability is unlikely the only cause of the varied response to acute caffeine ingestion as such effects were still prevalent in cases where between session reliability was good/excellent. For example, CMJ JH, PF, PP, and concentric impulse were highly reliable and demonstrated a main effect of treatment, However, when individual caffeine trials were compared to placebo, effect sizes varied between trivial to small. Given that experimental trials were conducted in identical conditions, it is speculated that the variation in response may be a related to participants mood, motivation, diet and prior activity, factors which may influence caffeine erogenicity.

As previously outlined, caffeine has been shown to effect mood (i.e., alertness, vigor, arousal, and decreased levels of fatigue [25,26,86] and motivation for exercise [25,26,28,29] which may mechanistically account for improved performance. Furthermore, the link between arousal and optimal performance have long been theorised [20]. Pre exercise RTE and FAS measured in the present study varied between sessions, and the poor between session reliability likely reflects inter-individual differences in mood and motivation that are difficult to standardise. The current findings therefore suggest that either caffeine elicits greater effects in certain mood states and/or an inability to standardise mood effects the ability to detect change (or lack of). 

Further contributing factors to the between session variation in caffeine’s effect may be attributable to fatigue state and pre-exercise diet. Although evidence is limited with respect to effects on physical performance, caffeine has been shown to be more effective for cognitive function following cognitive fatigue or sleep deprivation [87,88]. Whilst participants were instructed to abstain from intense physical activity 48 h prior to attendance, this was only verbally confirmed prior to each trial and sleep duration and quality were not measured. Similarly, food consumption, or lack of, has been shown to influence caffeine metabolism [89] which in turn may influence the ergogenic effect. Participants were instructed to abstain from caffeine at least 12 h prior to testing however this was only verbally confirmed and as such, the influence of diet on caffeine metabolism cannot be ruled out.

Although not without opposition [90], there is a suggestion that trained individuals may experience a greater caffeine induced effect [5,91] which has been attributed increased adenosine receptor density resulting in a greater potential to evoke an increased caffeine response [92]. It was considered that given the number of experimental trials and the nature of the exercise, training induced adaptation in muscular function over the course of the study may be in part responsible for the varied caffeine response seen in the results. However, this is unlikely to be the case given that the between trial comparison of caffeine effects compared to placebo did not show any distinct trend, nor was there any training effect as demonstrated in the post-trial reassessment of 1 RM. 

## 5. Limitations and Future Direction

The present study offers valuable insight into the repeatability of acute caffeine consumption on muscular strength and power performance but does not come without limitations. The ergogenic potential of caffeine has been shown to be influenced by variation in CYP1A2 and ADORA2A genes [80,93], factors that were not measured in the present study. Furthermore, measuring plasma concentration of caffeine would have been useful to ensure standardization at the start of each experimental trial. Furthermore, the results of the present study are only specific to participants with resistance training experience, where the impact of reliability on assessment measure may be greater in those that are naïve to the modes of exercise used in the present study.

It is clear from the present work, that if only a single caffeine trial was compared to single placebo trial, as is common in this field of work [2], a number of the conclusions regarding caffeine’s effect on strength would be different. Importantly, data in the present study provide novel insight into factors which may influence an ability to accurately detect caffeine effects and suggest a need to update standard practices evaluating caffeine ergogenicity. Specifically, there is a need for future research to explore multiple trials to allow detection of the potential fluctuation of effect from possible variations previously outlined (i.e., mood, motivation, diet, and fatigue) well as the chance of inter-individual responses to caffeine. This will allow further understanding of caffeine’s effect on strength and power to better understand how to maximize the ergogenic effect. 

## 6. Conclusions

The present study examined the repeatability of acute caffeine consumption of 3 mg.kg^−1^ effects on measures of muscular strength and power. Collectively these data demonstrate a contractile mode specific effect of caffeine on ingestion on specific measures of muscular strength and power. However, results from this study indicate for the first time that an individual is unlikely to see a performance enhancing benefit on every occasion where caffeine is consumed. Our data infer that benefits of caffeine ingestion may only be prevalent in athlete specific optimized mood and cognitive states. As such, athletes should seek to identify the condition where caffeine ingestion is likely to evoke the greatest performance enhancing benefits, and where this is not the case, consider the benefits of supplementation against well reported side effects. Furthermore, future work evaluating the ergogenic effects of caffeine on sports performance should assess response over multiple trials in order to more precisely evaluate its effects and prevent misleading results.

## Figures and Tables

**Table 1:** Acute effect of caffeine treatment (3 mg.kg^−1^) on readiness to invest effort physical, readiness to invest effort mental, and felt arousal.

		**Trial 1**			**Trial 2**			**Trial 3**			**Overall**			
		PL	CF	ES	PL	CF	ES	PL	CF	ES	PL	CF	ES	
RTE Phy	Pre	4.8 ± 2.7	4.5 ± 2.0	0.12	3.1 ± 1.8	4.6 ± 2.0	**0.77**	4.2 ± 2.3	4.6 ± 2.4	0.17	4.0 ± 2.4	4.6 ± 2.1	**0.26**	
	Post	5.1 ± 2.3	5.6 ± 1.7	**0.24**	3.9 ± 2.5	5.8 ± 1.7	**0.87**	5.4 ± 1.9	5.9 ± 1.8	**0.27**	4.8 ± 2.3	5.7 ± 1.7	**0.44**	
RTE Men	Pre	4.1 ± 2.4	4.7 ± 2.1	**0.26**	3.2 ± 1.9	4.0 ± 1.7	**0.44**	4.2 ± 2.1	4.5 ± 2.4	0.13	3.8 ± 2.2	4.4 ± 2.1	**0.27**	
	Post	4.8 ± 2.4	6.1 ± 1.8	**0.60**	4.1 ± 2.1	5.8 ± 1.6	**0.84**	4.7 ± 2.1	5.7 ± 1.8	0.11	4.8 ± 2.2	5.9 ± 1.7	**0.55**	
FAS	Pre	2 ± 1	3 ± 1	**0.98**	2 ± 1	2 ± 1	0.00	3 ± 1	3 ± 1	0.00	2 ± 1	2 ± 1	0.00	
	Post	3 ± 1	4 ± 1	**0.98**	3 ± 1	4 ± 1	**0.98**	2 ± 1	4 ± 1	0.98	3 ± 1	4 ± 1	**0.98**	

Note: Values are represented as means ± SD, PL= Placebo, CF= Caffeine, RTE Phy= Readiness to Invest Effort Physical, RTE Men= Readiness to Invest Effort Mental, FAS = Felt Arousal Scale, Pre = Pre-Treatment, Post = Post-Treatment, ES = Effect Size (Trivial > Bold Text).

**Table 2:** Effect of caffeine treatment (3 mg.kg^−1^) on CMJ performance.

		Trial 1	ES	Trial 2	ES	Trial 3	ES	Overall	ES
Jump Height (m)	PL	0.23 ± 0.07	**0.39**	0.23 ± 0.08	**0.39**	0.23 ± 0.06	0.16	0.23 ± 0.07	**0.28**
	CF	0.26 ± 0.08		0.26 ± 0.07		0.24 ± 0.06		0.25 ± 0.07	
RSI (Mod)	PL	0.33 ± 0.11	**0.47**	0.35 ± 0.14	0.14	0.34 ± 0.13	0.16	0.34 ± 0.13	**0.23**
	CF	0.39 ± 0.14		0.37 ± 0.14		0.36 ± 0.11		0.37 ± 0.13	
Peak Force (N.kg^−1^)	PL	14.8 ± 3.8	**0.39**	15.8 ± 3.8	0.08	15.7 ± 3.6	0.06	15.4 ± 3.7	0.17
	CF	16.2 ± 3.3		16.1 ± 3.8		15.9 ± 3.4		16.0 ± 3.4	
Peak Power (W.kg^−1^)	PL	43.2 ± 8.1	**0.40**	44.1 ± 8.8	0.08	43.1 ± 6.9	0.14	43.5 ± 7.9	**0.21**
	CF	46.7 ± 9.0		44.8 ± 8.0		44.1 ± 7.1		45.2. ± 8.0	
Concentric Impulse (Ns)	PL	180 ± 44	0.11	178 ± 47	0.08	181 ± 48	0.15	180 ± 46	0.11
	CF	185 ± 48		182 ± 47		188 ± 44		185 ± 46	
Eccentric Impulse (Ns)	PL	75 ± 22	0.04	77 ± 35	0.07	79 ± 36	0.03	77 ± 31	0.04
	CF	74 ± 23		75 ± 20		80 ± 25		76 ± 22	

Note: Values are represented as means ± SD, PL = Placebo, CF = Caffeine, and RSI (Mod) = Reactive Strength Index (Modified), ES = Effect Size (Trivial > Bold Text).

**Table 3:** Effect of caffeine treatment (3 mg.kg^−1^) on DJ performance.

		Trial 1	ES	Trial 2	ES	Trial 3	ES	Overall	ES
Jump Height (cm)	PL	0.30 ± 0.10	**0.28**	0.29 ± 0.10	**0.56**	0.30 ± 0.11	**0.29**	0.30 ± 0.10	**0.37**
	CF	0.33 ± 0.11		0.35 ± 0.11		0.33 ± 0.09		0.34 ± 0.11	
RSI	PL	0.91 ± 0.40	0.05	0.94 ± 0.38	0.03	0.95 ± 0.38	0.02	0.94 ± 0.38	0.03
	CF	0.93 ± 0.34		0.95 ± 0.39		0.96 ± 0.44		0.95 ± 0.39	
Peak Force (N.Kg)	PL	51.9 ± 10.2	0.10	51.0 ± 9.6	**0.42**	50.2 ± 11.5	**0.21**	51.0 ± 0.10.3	**0.24**
	CF	53.0 ± 11.4		55.3 ± 10.6		52.8 ± 12.7		53.7 ± 0.11.5	
Peak Power (W.Kg)	PL	67.8 ± 20.7	**0.39**	65.5 ± 17.2	**0.55**	65.2 ± 21.0	**0.25**	66.2 ± 0.19.4	**0.40**
	CF	78.6 ± 32.1		77.4 ± 24.5		70.4 ± 19.3		75.5 ± 0.25.7	

Note: Values are represented as means ± SD, PL = Placebo, CF = Caffeine, and RSI= Reactive Strength Index, ES = Effect Size (Trivial > Bold Text).

**Table 4:** Effect of caffeine treatment (3 mg.kg^−1^) on IMTP performance.

		Trial 1	ES	Trial 2	ES	Trial 3	ES	Overall	ES
Peak force (N.Kg)	PL	27.8 ± 7.8	**0.32**	25.0 ± 4.1	**0.34**	25.8 ± 5.2	0.08	26.2 ± 5.9	0.00
	CF	25.7 ± 4.9		26.6 ± 5.2		26.2 ± 4.5		26.2 ± 4.8	
Time to peak Force (Sec)	PL	2.50 ± 1.58	**0.32**	2.62 ± 1.54	**0.29**	2.15 ± 1.09	0.10	2.42 ± 1.41	**0.25**
	CF	2.02 ± 1.37		2.21 ± 1.27		2.02 ± 1.34		2.08 ± 1.31	
Force (N) at 100 m/s	PL	1280 ± 302	0.05	1281 ± 346	**0.22**	1277 ± 417	0.18	1285 ± 347	0.15
	CF	1299 ± 417		1356 ± 319		1346 ± 376		1342 ± 375	
Force (N) at 300 m/s	PL	1858 ± 461	0.02	1817 ± 583	0.02	1877 ± 583	0.08	1863 ± 487	0.03
	CF	1867 ± 475		1825 ± 486		1916 ± 400		1879 ± 458	

Note: Values are represented as means ± SD, PL = Placebo, CF = Caffeine, ES = Effect Size (Trivial < Bold Text).

**Table 5:** Effect of caffeine treatment (3 mg.kg^−1^) on upper and lower body resistance exercise; chest press, shoulder press, squats, and deadlift reps until failure as well as chest press, shoulder press, squats, and deadlift total weight lifted (kg).

		Trial 1			Trial 2			Trial 3			*Overall*		
		PL	CF	ES	PL	CF	ES	PL	CF	ES	*PL*	*CF*	ES
CP	Set1	13 ± 3	14 ± 5	**0.24**	13 ± 5	14 ± 4	**0.43**	14 ± 6	14 ± 4	0.00	13 ± 4	14 ± 4	**0.25** **0.25**
	Set2	10 ± 3	14 ± 4	**0.83**	11 ± 4	12 ± 4	**0.25**	11 ± 4	12 ± 4	**0.25**	11 ± 4	12 ± 4	
SP	Set1	12 ± 3	13 ± 4	**0.28**	10 ± 3	12 ± 4	**0.56**	12 ± 3	13 ± 4	**0.28**	11 ± 3	13 ± 4	**0.56** **0.65**
	Set2	9 ± 3	11 ± 3	**0.65**	9 ± 3	10 ± 3	**0.33**	10 ± 3	11 ± 2	**0.39**	9 ± 3	11 ± 3	
SQ	Set1	13 ± 4	15 ± 5	**0.43**	12 ± 4	16 ± 4	**0.98**	14 ± 4	16 ± 5	**0.43**	13 ± 4	15 ± 5	**0.43** **0.25**
	Set2	12 ± 4	13 ± 4	**0.25**	11 ± 4	13 ± 4	**0.49**	12 ± 3	13 ± 4	**0.28**	12 ± 4	13 ± 4	
DL	Set1	12 ± 4	14 ± 6	**0.39**	11 ± 4	13 ± 4	**0.49**	12 ± 5	14 ± 7	**0.32**	12 ± 5	13 ± 6	0.18 **0.25**
	Set2	10 ± 4	11 ± 4	**0.25**	9 ± 4	11 ± 4	**0.49**	10 ± 3	11 ± 3	**0.33**	10 ± 4	11 ± 4	

Note: Values are represented as means ± SD, PL = Placebo, CF = Caffeine, CP = Chest Press, SP = Shoulder Press, SQ = Squats, DL = Deadlift, ES = Effect Size (Trivial < Bold Text).

**Table 6:** Effect of caffeine treatment (3 mg.kg^−1^) on RPE following repetitions until failure protocol.

		Trial 1			Trial 2			Trial 3			Overall		
		PL	CF	ES	PL	CF	ES	PL	CF	ES	PL	CF	ES
CP	Set1	17 ± 1	18 ± 1	**0.98**	18 ± 2	18 ± 1	0.00	17 ± 1	18 ± 2	**0.62**	17 ± 1	18 ± 1	**0.98** **0.98**
	Set2	19 ± 1	18 ± 1	**0.98**	18 ± 1	19 ± 1	**0.98**	18 ± 1	19 ± 1	**0.98**	19 ± 1	18 ± 1	
SP	Set1	17 ± 1	18 ± 1	**0.98**	18 ± 2	18 ± 1	0.00	17 ± 1	18 ± 2	**0.62**	18 ± 1	17 ± 2	**0.62** 0.00
	Set2	19 ± 1	18 ± 1	**0.98**	18 ± 1	19 ± 1	**0.98**	18 ± 1	19 ± 1	**0.98**	18 ± 1	18 ± 1	
SQ	Set1	17 ± 1	17 ± 1	0.00	18 ± 1	17 ± 1	**0.98**	17 ± 1	18 ± 1	**0.98**	17 ± 1	17 ± 1	0.00 **0.98**
	Set2	18 ± 1	18 ± 1	0.00	19 ± 1	18 ± 1	**0.98**	18 ± 1	19 ± 1	**0.98**	19 ± 1	18 ± 1	
DL	Set1	17 ± 2	17 ± 1	0.00	17 ± 2	18 ± 3	**0.62**	18 ± 1	18 ± 1	0.00	17 ± 2	18 ± 1	**0.62** 0.00
	Set2	18 ± 3	19 ± 1	**0.44**	18 ± 1	18 ± 1	0.00	19 ± 1	18 ± 1	**0.98**	18 ± 2	18 ± 1	

Note: Values are represented as means ± SD, PL = Placebo, CF = Caffeine, CP = Chest Press, SP = Shoulder Press, SQ= Squats, DL= Deadlift, ES = Effect Size (Trivial < Bold Text).

## Data Availability

Not applicable.

## Supplementary Materials

The following supporting information can be downloaded at: https://www.mdpi.com/article/10.3390/nu14204416/s1, Table S1: Between trial test-retest reliability of readiness to invest physically, readiness to invest effort mentally, and felt arousal pre-and post-treatment; Table S2: Between trial test-retest reliability of CMJ performance; Table S3: Between trial test-retest reliability of DJ performance; Table S4: Between trial test-retest reliability of IMTP performance; Table S5: Between trial test-retest reliability of repetitions until failure during Chest Press, Shoulder Press, Squats, and Deadlifts; Table S6: Between trial test-retest reliailbity of rate of perceived exertion following repetitions until failure protocol.

## References

[1] Warren G.L., Park N.D., Maresca R.D., McKibans K.I., Millard-Stafford M.L. (2010). Effect of caffeine ingestion on muscular strength and endurance: A meta-analysis. Med. Sci. Sports Exerc..

[2] Grgic J., Grgic I., Pickering C., Schoenfeld B.J., Bishop D.J., Pedisic Z. (2020). Wake up and smell the coffee: Caffeine supplementation and exercise performance—An umbrella review of 21 published meta-analyses. Br. J. Sports Med..

[3] Burke L.M. (2008). Caffeine and sports performance. Appl. Physiol. Nutr. Metab..

[4] Astorino T.A., Terzi M.N., Roberson D.W., Burnett T.R. (2010). Effect of two doses of caffeine on muscular function during isokinetic exercise. Med. Sci. Sports Exerc..

[5] Grgic J., Trexler E.T., Lazinica B., Pedisic Z. (2018). Effects of caffeine intake on muscle strength and power: A systematic review and meta-analysis. J. Int. Soc. Sports Nutr..

[6] Polito M.D., Souza D.B., Casonatto J., Farinatti P. (2016). Acute effect of caffeine consumption on isotonic muscular strength and endurance: A systematic review and meta-analysis. Sci. Sports.

[7] Grgic J., Pickering C. (2019). The effects of caffeine ingestion on isokinetic muscular strength: A meta-analysis. J. Sci. Med. Sport.

[8] Southward K., Rutherfurd-Markwick K.J., Ali A. (2018). The effect of acute caffeine ingestion on endurance performance: A systematic review and meta–analysis. Sports Med..

[9] Salinero J.J., Lara B., Del Coso J. (2019). Effects of acute ingestion of caffeine on team sports performance: A systematic review and meta-analysis. Res. Sports Med..

[10] Glaister M., Gissane C. (2018). Caffeine and physiological responses to submaximal exercise: A meta-analysis. Int. J. Sports Physiol. Perform..

[11] Ribeiro B.G., Morales A.P., Sampaio-Jorge F., de Souza Tinoco F., de Matos A.A., Leite T.C. (2017). Acute effects of caffeine intake on athletic performance: A systematic review and meta-analysis. Revista Chilena de Nutrición.

[12] Glaister M., Moir G. (2019). Effects of caffeine on time trial performance and associated physiological responses: A meta-analysis. J. Caffeine Adenosine Res..

[13] Tallis J., Duncan M.J., James R.S. (2015). What can isolated skeletal muscle experiments tell us about the effects of caffeine on exercise performance?. Br. J. Pharmacol..

[14] Pickering C., Kiely J. (2018). Are the current guidelines on caffeine use in sport optimal for everyone? Inter-individual variation in caffeine ergogenicity, and a move towards personalised sports nutrition. Sports Med..

[15] Grgic J., Mikulic P., Schoenfeld B.J., Bishop D.J., Pedisic Z. (2019). The influence of caffeine supplementation on resistance exercise: A review. Sports Med..

[16] Astorino T.A., Rohmann R.L., Firth K. (2008). Effect of caffeine ingestion on one-repetition maximum muscular strength. Eur. J. Appl. Physiol..

[17] Ali A., O’Donnell J., Foskett A., Rutherfurd-Markwick K. (2016). The influence of caffeine ingestion on strength and power performance in female team-sport players. J. Int. Soc. Sports Nutr..

[18] Grgic J., Mikulic P. (2017). Caffeine ingestion acutely enhances muscular strength and power but not muscular endurance in resistance-trained men. Eur. J. Sport Sci..

[19] Souza A.A., Bottaro M., Rocha V.A., Lage V., Tufano J.J., Vieira A. (2020). Reliability and test-retest agreement of mechanical variables obtained during countermovement jump. Int. J. Exerc. Sci..

[20] Perkins D., Wilson G.V., Kerr J.H. (2001). The effects of elevated arousal and mood on maximal strength performance in athletes. J. Appl. Sport Psychol..

[21] Roberts G.C., Nerstad C.G., Lemyre P.N. (2018). Motivation in Sport and Performance.

[22] Teo W., Newton M.J., McGuigan M.R. (2011). Circadian rhythms in exercise performance: Implications for hormonal and muscular adaptation. J. Sports Sci. Med..

[23] Fullagar H.H., Skorski S., Duffield R., Hammes D., Coutts A.J., Meyer T. (2015). Sleep and athletic performance: The effects of sleep loss on exercise performance, and physiological and cognitive responses to exercise. Sports Med..

[24] Knowles O.E., Drinkwater E.J., Urwin C.S., Lamon S., Aisbett B. (2018). Inadequate sleep and muscle strength: Implications for resistance training. J. Sci. Med. Sport.

[25] Duncan M.J., Oxford S.W. (2011). The effect of caffeine ingestion on mood state and bench press performance to failure. J. Strength Cond. Res..

[26] Duncan M.J., Smith M., Cook K., James R.S. (2012). The acute effect of a caffeine-containing energy drink on mood state, readiness to invest effort, and resistance exercise to failure. J. Strength Cond. Res..

[27] Harrell P.T., Juliano L.M. (2009). Caffeine expectancies influence the subjective and behavioral effects of caffeine. Psychopharmacology.

[28] Tallis J., Muhammad B., Islam M., Duncan M.J. (2016). Placebo effects of caffeine on maximal voluntary concentric force of the knee flexors and extensors. Muscle Nerve.

[29] Childs E., de Wit H. (2008). Enhanced mood and psychomotor performance by a caffeine-containing energy capsule in fatigued individuals. Exp. Clin. Psychopharmacol..

[30] Pickering C., Kiely J. (2019). Are low doses of caffeine as ergogenic as higher doses? A critical review highlighting the need for comparison with current best practice in caffeine research. Nutrition.

[31] Southward K., Rutherfurd-Markwick K., Badenhorst C., Ali A. (2018). The role of genetics in moderating the inter-individual differences in the ergogenicity of caffeine. Nutrients.

[32] Black C.D., Waddell D.E., Gonglach A.R. (2015). Caffeine’s Ergogenic Effects on Cycling: Neuromuscular and Perceptual Factors. Med. Sci. Sports Exerc..

[33] Tallis J., Yavuz H.C. (2018). The effects of low and moderate doses of caffeine supplementation on upper and lower body maximal voluntary concentric and eccentric muscle force. Appl. Physiol. Nutr. Metab..

[34] Timmins T.D., Saunders D.H. (2014). Effect of caffeine ingestion on maximal voluntary contraction strength in upper-and lower-body muscle groups. J. Strength Cond. Res..

[35] Grgic J., Schoenfeld B.J., Davies T.B., Lazinica B., Krieger J.W., Pedisic Z. (2018). Effect of resistance training frequency on gains in muscular strength: A systematic review and meta-analysis. Sports Med..

[36] Ferreira T.T., da Silva J.V.F., Bueno N.B. (2021). Effects of caffeine supplementation on muscle endurance, maximum strength, and perceived exertion in adults submitted to strength training: A systematic review and meta-analyses. Crit. Rev. Food Sci. Nutr..

[37] Shohet K.L., Landrum R.E. (2001). Caffeine consumption questionnaire: A standardized measure for caffeine consumption in undergraduate students. Psychol. Rep..

[38] Graham T.E. (2001). Caffeine and exercise. Sports Med..

[39] Tamilio R.A., Clarke N.D., Duncan M.J., Morris R., Grgic J., Tallis J. (2021). Can 3 mg·kg^−1^ of Caffeine Be Used as An Effective Nutritional Supplement to Enhance the Effects of Resistance Training in Rugby Union Players?. Nutrients.

[40] Baechle T.R., Earle R.W. (2008). Essentials of Strength Training and Conditioning.

[41] Maud P.J., Foster C. (2006). Strength testing: Development and Evaluation of Methodology. Physiological Assessment of Human Fitness.

[42] Grgic J., Lazinica B., Schoenfeld B.J., Pedisic Z. (2020). Test-retest reliability of the one-repetition maximum (1RM) strength assessment: A systematic review. Sports Med. Open.

[43] McMahon J.J., Suchomel T.J., Lake J.P., Comfort P. (2018). Understanding the key phases of the countermovement jump force-time curve. Strength Cond. J..

[44] Chavda S., Bromley T., Jarvis P., Williams S., Bishop C., Turner A.N., Lake J.P., Mundy P.D. (2018). Force-time characteristics of the countermovement jump: Analyzing the curve in Excel. Strength Cond. J..

[45] Moir G.L. (2008). Three different methods of calculating vertical jump height from force platform data in men and women. Meas. Phys. Educ. Exerc. Sci..

[46] Boukhenous S., Attari M. A Vertical Jumping Performance with and without Arms Swing by Using a Dynamometric Platform. Proceedings of the International Workshop on Systems, Signal Processing and their Applications, WOSSPA.

[47] Wade L., Needham L., McGuigan M.P., Bilzon J.L. (2022). Backward Double Integration is a Valid Method to Calculate Maximal and Sub-Maximal Jump Height. J. Sports Sci..

[48] Makaruk H., Sacewicz T., Czaplicki A., Sadowski J. (2010). Effect of additional load on power output during drop jump training. J. Hum. Kinet..

[49] Dos’ Santos T., Thomas C., Comfort P., McMahon J.J., Jones P.A., Oakley N.P., Young A.L. (2018). Between-session reliability of isometric midthigh pull kinetics and maximal power clean performance in male youth soccer players. J. Strength Cond. Res..

[50] Morris R.O., Jones B., Myers T., Lake J., Emmonds S., Clarke N.D., Singleton D., Ellis M., Till K. (2020). Isometric midthigh pull characteristics in elite youth male soccer players: Comparisons by age and maturity offset. J. Strength Cond. Res..

[51] Haff G.G., Ruben R.P., Lider J., Twine C., Cormie P. (2015). A comparison of methods for determining the rate of force development during isometric midthigh clean pulls. J. Strength Cond. Res..

[52] Kuki S., Sato K., Stone M.H., Okano K., Yoshida T., Tanigawa S. (2017). The relationship between isometric mid-thigh pull variables, jump variables and sprint performance in collegiate soccer players. J. Trainology.

[53] Dos’ Santos T., Jones P.A., Comfort P., Thomas C. (2017). Effect of different onset thresholds on isometric midthigh pull force-time variables. J. Strength Cond. Res..

[54] Mundy P.D., Lake J.P., Carden P.J., Smith N.A., Lauder M.A. (2016). Agreement between the force platform method and the combined method measurements of power output during the loaded countermovement jump. Sports Biomech..

[55] Duncan M.J., Stanley M., Parkhouse N., Cook K., Smith M. (2013). Acute caffeine ingestion enhances strength performance and reduces perceived exertion and muscle pain perception during resistance exercise. Eur. J. Sport Sci..

[56] Duncan M.J., Oxford S.W. (2012). Acute caffeine ingestion enhances performance and dampens muscle pain following resistance exercise to failure. J. Sports Med. Phys. Fit..

[57] Martorelli S., Cadore E.L., Izquierdo M., Celes R., Martorelli A., Cleto V.A., Alvarenga J.G., Bottaro M. (2017). Strength training with repetitions to failure does not provide additional strength and muscle hypertrophy gains in young women. Eur. J. Transl. Myol..

[58] American College of Sports Medicine (2009). American College of Sports Medicine position stand. Progression models in resistance training for healthy adults. Med. Sci. Sports Exerc..

[59] Borg G.A. (1982). Psychophysical bases of perceived exertion. Med. Sci. Sports Exerc..

[60] Fredholm B.B., Bättig K., Holmén J., Nehlig A., Zvartau E.E. (1999). Actions of caffeine in the brain with special reference to factors that contribute to its widespread use. Pharmacol. Rev..

[61] Raedeke T.D., Focht B.C., Scales D. (2009). Mediators of affective responses to acute exercise among women with high social physique anxiety. Psychol. Sport Exerc..

[62] Noble S., Scheinost D., Constable R.T. (2019). A decade of test-retest reliability of functional connectivity: A systematic review and meta-analysis. Neuroimage.

[63] Abdi H., Salkind N. (2010). Coefficient of Variation. Encyclopedia of Research Design.

[64] Bunce C. (2009). Correlation, agreement, and Bland–Altman analysis: Statistical analysis of method comparison studies. Am. J. Ophthalmol..

[65] Bland J.M., Altman D. (1986). Statistical methods for assessing agreement between two methods of clinical measurement. Lancet.

[66] Tabachnick B.G., Fidell L.S., Ullman J.B. (2007). Using Multivariate Statistics.

[67] Richardson J.T. (2011). Eta squared and partial eta squared as measures of effect size in educational research. Educ. Res. Rev..

[68] Hedges L.V. (1981). Distribution theory for Glass’s estimator of effect size and related estimators. J. Educ. Stat..

[69] Cohen J. (2013). Statistical Power Analysis for the Behavioral Sciences.

[70] Burke B.I., Travis S.K., Gentles J.A., Sato K., Lang H.M., Bazyler C.D. (2021). The effects of caffeine on jumping performance and maximal strength in female collegiate athletes. Nutrients.

[71] Harty P.S., Zabriskie H.A., Stecker R.A., Currier B.S., Tinsley G.M., Surowiec K., Jagim A.R., Richmond S.R., Kerksick C.M. (2020). Caffeine timing improves lower-body muscular performance: A randomized trial. Front. Nutr..

[72] Astorino T.A., Roberson D.W. (2010). Efficacy of acute caffeine ingestion for short-term high-intensity exercise performance: A systematic review. J. Strength Cond. Res..

[73] Da Silva V.L., Messias F.R., Zanchi N.E., Gerlinger-Romero F., Duncan M.J., Guimarães-Ferreira L. (2015). Effects of acute caffeine ingestion on resistance training performance and perceptual responses during repeated sets to failure. J. Sports Med. Phys. Fit..

[74] Aragón-Vela J., Casuso R.A., Casals C., Plaza-Díaz J., Fontana L., Huertas J.R. (2020). Differential IL 10 serum production between an arm-based and a leg-based maximal resistance test. Cytokine.

[75] Arazi H., Sotoudeh K., Sadeghi M.M., Mohammadi S.M., Saeedi T. (2015). Influence of Upper-Body Exercise Order on Repetition Performance and Ratings of Perceived Exertion During A Super-Set Resistance Training Session. J. Sport Sci..

[76] de Azevedo A.P., Guerra M.A., Caldas L.C., Guimarães-Ferreira L. (2019). Acute caffeine ingestion did not enhance punch performance in professional mixed-martial arts athletes. Nutrients.

[77] Tallis J., Duncan M.J., Wright S.L., Eyre E.L., Bryant E., Langdon D., James R.S. (2013). Assessment of the ergogenic effect of caffeine supplementation on mood, anticipation timing, and muscular strength in older adults. Physiol. Rep..

[78] Shabir A., Hooton A., Tallis J., Higgins M.F. (2018). The influence of caffeine expectancies on sport, exercise, and cognitive performance. Nutrients.

[79] Williams A.D., Cribb P.J., Cooke M.B., Hayes A. (2008). The effect of ephedra and caffeine on maximal strength and power in resistance-trained athletes. J. Strength Cond. Res..

[80] Tennent R., Ali A., Wham C., Rutherfurd-Markwick K. (2020). Narrative Review: Impact of Genetic Variability of CYP1A2, ADORA2A, and AHR on Caffeine Consumption and Response. J. Caffeine Adenosine Res..

[81] Brady C.J., Harrison A.J., Comyns T.M. (2018). A review of the reliability of biomechanical variables produced during the isometric mid-thigh pull and isometric squat and the reporting of normative data. Sports Biomech..

[82] Moeskops S., Oliver J.L., Read P.J., Cronin J.B., Myer G.D., Haff G.G., Lloyd R.S. (2018). Within-and between-session reliability of the isometric mid-thigh pull in young female athletes. J. Strength Cond. Res..

[83] Heishman A.D., Daub B.D., Miller R.M., Freitas E.D., Frantz B.A., Bemben M.G. (2020). Countermovement jump reliability performed with and without an arm swing in NCAA division 1 intercollegiate basketball players. J. Strength Cond. Res..

[84] Merrigan J.J., Stone J.D., Hornsby W.G., Hagen J.A. (2020). Identifying reliable and relatable force–time metrics in athletes—Considerations for the isometric mid-thigh pull and countermovement jump. Sports.

[85] Grgic J., Scapec B., Mikulic P., Pedisic Z. (2021). Test-retest reliability of isometric mid-thigh pull maximum strength assessment: A systematic review. Biol. Sport.

[86] Huntley E.D., Juliano L.M. (2012). Caffeine Expectancy Questionnaire (CaffEQ): Construction, psychometric properties, and associations with caffeine use, caffeine dependence, and other related variables. Psychol. Assess..

[87] Snel J., Lorist M.M. (2011). Effects of caffeine on sleep and cognition. Prog. Brain Res..

[88] Roehrs T., Roth T. (2008). Caffeine: Sleep and daytime sleepiness. Sleep Med. Rev..

[89] Nehlig A. (2018). Interindividual differences in caffeine metabolism and factors driving caffeine consumption. Pharmacol. Rev..

[90] Brooks J.H., Wyld K., Chrismas B.C. (2015). Acute effects of caffeine on strength performance in trained and untrained individuals. J. Athl. Enhanc..

[91] Astorino T.A., Cottrell T., Lozano A.T., Aburto-Pratt K., Duhon J. (2012). Effect of caffeine on RPE and perceptions of pain, arousal, and pleasure/displeasure during a cycling time trial in endurance trained and active men. Physiol. Behav..

[92] Pickering C., Kiely J. (2019). What should we do about habitual caffeine use in athletes?. Sports Med..

[93] Fulton J.L., Dinas P.C., Carrillo A.E., Edsall J.R., Ryan E.J., Ryan E.J. (2018). Impact of genetic variability on physiological responses to caffeine in humans: A systematic review. Nutrients.

