# Supplementary material for: How Repeatable Is the Ergogenic Effect of Caffeine? Limited Reproducibility of Acute Caffeine (3 mg.kg−1) Ingestion on Muscular Strength, Power, and Muscular Endurance
Source: Nutrients. 2022 Oct 21;14(20):4416. doi: 10.3390/nu14204416 (PMC9611362; doi:10.3390/nu14204416)
Supplement: Supplementary file 1 [file nutrients-14-04416-s001.zip › nutrients-1971077-supplementary.pdf]

Supplementary Table 1: Between trial test-retest reliability of readiness to invest effort physically, readiness to invest effort mentally, and felt arousal pre-and post-treatment.

		Placebo				Caffeine			
		PL Trials	T1 vs T2	T1 vs T3	T2 vs T3	CF Trials	T1 vs T2	T1 vs T3	T2 vs T3
RTE Phy (Pre)	ICC	.521	.373	.722	.259	.619	.607	.545	.734
	95% CI	.267-.740	-.022-.676	.445-.874	-.125-.593	.387-.802	.252-.816	.162-.783	.456-.880
	Significance	.001*	.013*	.001*	.096	.001*	.001*	.004*	.000*
	CV	37.6%	44.2%	29.9%	38.7%	24.4%	21.4%	25%	26.8%
	Mean bias		1.7	0.6	-1.0		-0.1	-0.2	-0.1
	Lo-Up LOA		-3.0-6.3	-3.0-4.2	-6.0-3.9		-3.6-3.4	-4.3-4.0	-3.2-3.1
RTE Phy (Post)	ICC	.258	.269	.489	.001	.147	.329	-.012	.162
	95% CI	.001-.543	-.144-.600	.094-.751	-.322-.371	.092-.444	-.109-.656	-.448-.414	-.292-.546
	Significance	.024*	.086	.010*	.498	.124	.068	.521	.239
	CV	36.4%	40.6%	26.1%	42.4%	20.4%	19.9%	20.4%	20.8%
	Mean bias		1.2	-0.3	-1.5		-0.2	-0.3	-0.1
	Lo-Up LOA		-4.4-6.8	-4.6-3.9	-7.6-4.6		-4.1-3.7	-5.1-4.4	-4.9-4.6
RTE Men (Pre)	ICC	.504	.546	.589	.294	.381	.394	.547	.171
	95% CI	.249-729	.182-.781	.225-.807	-.090-.618	.117-641	.002-.689	.168-.784	-.263-.546
	Significance	.001*	.002*	.002*	.068	.002*	.026*	.004*	.220
	CV	36.8%	36.9%	35.9%	37.5%	31.7%	30.7%	30.1%	34.3%
	Mean bias		0.9	-0.1	-1.0		0.7	0.2	-0.5
	Lo-Up LOA		-2.9-4.7	-4.2-4.0	-5.6-3.6		-3.4-4.8	-4.1-4.5	-5.8-4.8
RTE Men (Post)	ICC	.240	.310	.368	.090	.321	.586	.115	.299
	95% CI	.014-528	-.104-.637	.049-.677	-.267-.458	.059-595	.228-.805	-.324.508	-.146-.638
	Significance	.033*	.072	.042*	.322	.007*	.002*	.304	.089
	CV	35.6%	35.7%	32.2%	38.9%	20.5%	16.9%	24.9%	19.6%
	Mean bias		0.7	-0.7	-1.4		0.2	0.4	0.1
	Lo-Up LOA		-4.7-6.1	-5.4-4.0	-7.0-4.2		-2.9-3.3	-4.4-5.1	-3.9-4.2
FAS (Pre)	ICC	.564	.474	.695	.382	.587	.755	.559	.454
	95% CI	.319-.768	.096-.738	.393-.861	.002-.679	.346-.782	.500-.890	.184-.791	.038-.732
	Significance	.001*	.006*	.001*	.017*	.001*	.001*	.003*	.017*
	CV	23.7%	24.8%	19.6%	26.6%	20.2%	16.2%	24%	20.4%

	Mean bias		1	0	-1		1	0	0
	Lo-Up LOA		-2 - 3	-2 - 2	-3 – 1		-1 - 2	-2 - 2	-2 - 2
FAS (Post)	ICC	.377	.288	.500	.287	.366	.520	.119	.475
	95% CI	.114-.639	.110-.618	.112-.757	.079-.608	.103-.630	.129-.769	-.334-.515	.068-.744
	Significance	.002*	.080	.008*	.057	.003*	.006*	.302	.013*
	CV	24.3%	25.8%	20.3%	26.9%	15.8%	12%	22.2%	13.3%
	Mean bias		1	0	1		0	0	0
	Lo-Up LOA		-3 - 4	-3 - 2	-2 - 4		-2 - 2	-3 - 3	-2 - 2

Note: RTE Phy= Readiness to Invest Effort Physical, RTE Men= Readiness to Invest Effort Mental, FAS= Felt Arousal, T1= Trial 1, T2= Trial 2, T3= Trial 3, ICC= Intra-class correlation, 95% CI= 95% confidence interval, CV= Coefficient of variation, and Lo-Up LOA= Lower and Upper 95% limits of agreement, Effect Size = Trivial < Bold Text, *= P<0.05.

Effect of Caffeine on Countermovement Jump Performance

Supplementary Table 2: Between trial test-retest reliability of CMJ performance

		Placebo				Caffeine			
		PL Trials	T1 vs T2	T1 vs T3	T2 vs T3	CF Trials	T1 vs T2	T1 vs T3	T2 vs T3
Jump height (m)	ICC	.870	.893	.841	.881	.786	.745	.805	.767
	95% CI	.757-939	.760-.954	.656-.931	.735-.949	.621-896	.475-.886	.555-.917	.485-.900
	Significance	.001*	.001*	. 001	.001*	.001*	.001*	.001*	.001*
	CV	9.2%	8.6%	9.9%	9.1	11.3%	13.3%	9%	11.6%
	Mean bias		.003	.004	.001		.001	.021	.021
	Lo-Up LOA		-.07-.07	-.07-.08	-.07-.07		-.11-.11	-.06-.10	-.06-.10
RSI (Mod)	ICC	.680	.716	.683	.660	.793	.761	.823	.799
	95% CI	.467-.838	.436-870	.378-.855	.335-.844	.631-.900	.509-.893	.619-923	.578-911
	Significance	.001*	.001*	.001*	.001*	.001*	.001*	.001*	.001*
	CV	15.2%	13.7%	18.1%	12.6%	12.7%	16.4%	11.8%	10.4%
	Mean bias		-.020	-.015	.003		.018	.028	.009
	Lo-Up LOA		-.20-.16	-.21-.18	-.23-.23		-.17-.21	-.11-.16	-.13-.15
Peak Force (NKg-1)	ICC	.877	.838	.880	.882	.752	.724	.767	.791
	95% CI	770-.942	.629-.932	.676-.952	.737-949	.569-.878	.439-.876	.520-896	.561-908
	Significance	.001*	.001*	. 001*	.001*	.001	.001*	.001*	.001*
	CV	8.0%	9.2%	7.9%	6.9%	8.2%	8.3%	8%	8.2%
	Mean bias		-.94	-.92	.02		.08	.29	.21

	Lo-Up LOA		-4.9-3.0	-4.1-2.3	-3.6-3.6		-5.2-5.3	-4.2-4.8	-4.4-4.8
Peak Power (WKg-1)	ICC	.881	.931	.811	.901	.745	.808	.833	.893
	95% CI	.777-.944	.843-.971	.602-.917	.776-.958	.560-.874	.599-.915	.645-.927	.760-.954
	Significance	.001*	.001*	.001*	.001*	.001*	.001*	.001*	.001*
	CV	5.4%	5.2%	6%	5%	6.4%	7.1%	5.9%	6.2%
	Mean bias		-.87	0.13	1.01		1.90	2.64	0.74
	Lo-Up LOA		-8.2-6.5	-8.3-8.6	-6.1-8.1		-10.7-14.5	-9.0-14.3	-8.7-10.2
Con Impulse (Ns)	ICC	.857	.909	.770	.907	.917	.913	.934	.905
	95% CI	.736-.933	.794-.961	.521-.898	.790-.960	.841-.962	.804-.963	.850-.972	.768-.959
	Significance	.001*	.001*	.001*	.001*	.001*	.001*	.001*	.001*
	CV	5.5%	5.5%	6.6%	5.5%	5.5%	5.3%	5.5%	6.8%
	Lo-Up LOA		-37-40	-63-62	-43-39		-37-42	-36-30	-44-33
Ecc Impulse (Ns)	ICC	.815	.711	.762	.931	.866	.873	.852	.841
	95% CI	.667-.911	.420-.869	.514-.893	.842-.971	.750-.937	.719-.945	.649-.938	.649-932
	Significance	.001*	.001*	.001*	.001*	.001*	.001*	.001*	.001*
	CV	10.2%	9.9%	11.2%	10.2%	8.8%	7.8%	8.8%	10%
	Mean bias		-2	-4	-2		-1	-6	-5
	Lo-Up LOA		-46-42	-44-36	-28-25		-22-20	-30-18	-29-19

Note: Con= Concentric, Ecc= Eccentric, T1= Trial 1, T2= Trial 2, T3= Trial 3, RSI (mod)= Reactive Strength Index (modified) ICC= Interclass correlation, 95% CI= 95% confidence interval, CV= Coefficient of variation, Lo-Up LOA= Lower and Upper 95% limits of agreement, Effect Size = Trivial < Bold Text * = P< 0.05.

Effect of Caffeine on Drop Jump Performance

Supplementary Table 3: Between trial test-retest reliability of DJ performance

		Placebo				Caffeine			
		PL Trials	T1 vs T2	T1 vs T3	T2 vs T3	CF Trials	T1 vs T2	T1 vs T3	T2 vs T3
Jump height (m)	ICC	.313	.175	.265	.468	.475	.470	.300	.657
	95% CI	.052-.589	-.257-.549	-.166-.612	.091-.749	.216-.709	.071-.740	-129.-.634	.334-.841
	Significance	.009*	.212	.110	.009*	.001*	.012*	.082	.001*
	CV	21.5%	23.9%	22.1%	18.5%	17.7%	20.2%	19.7%	13.3%
	Mean bias		.005	.002	-.007		-.025	-.004	.021
	Lo-Up LOA		-.26-.27	-.26 - .25	-.22-.21		-.25-.21	-.24–.23	-.15.19
RSI	ICC	.902	.865	.886	.957	.902	.857	.899	.941

	95% CI	.813-.954	.703-.942	.746-.951	.899-.982	.814-.955	.687-.938	.772-957.	.863-975.
	Significance	.001*	.001*	.001*	.001*	.001*	.001*	.001*	.001*
	CV	10.3%	12.1%	11.7%	7%	9.3%	10.6%	9.7%	7.8%
	Mean bias		-.031	-.043	-012		-.015	-.027	-.013
	Lo-Up LOA		-.43 -.36	-.41 –.32	-23 -.21		-.40 -.37	-.38 –.32	-.29 -.27
Peak Force (N. Kg)	ICC	.600	.598	.665	.531	.543	.471	.580	.568
	95% CI	.362-.790	.246-.811	.348-.846	.151-.774	.294-.754	.071-.740	.219-.801	.203-.795
	Significance	.001*	.001*	.001*	.005*	.001*	.012*	.002*	.002*
	CV	9.9%	9.2%	9.7%	10.8%	12.1%	12.9%	12.1%	11.2%
	Mean bias		.99	1.76	.77		-2.37	-.18	2.55
	Lo-Up LOA		-16.3-18.3	-15.6-19.2	-19.3-20.9		-24.6-19.8	-21.5-21.8	-18.7- 23.8
Peak Power (W. Kg)	ICC	.388	.258	.351	.559	.421	.435	.450	.441
	95% CI	.125-.647	-.174-.607	-.072-.668	.190-.790	.158-.671	.026-.719	.046-.728	.034-.723
	Significance	.002*	.117	.050*	.003*	.001*	.019*	.016*	.018*
	CV	21.2%	21.5%	26%	16%	20.9%	25.1%	22%	15.7%
	Mean bias		2.27	-13.10	0.30		1.18	8.21	7.03
	Lo-Up LOA		-43.1-47.7	-71.6-45.4	-35.0-35.6		-58.3-60.6	-46.2-62.7	-38.7-52.7

Note: T1= Trial 1, T2= Trial 2, T3= Trial 3, RSI= Reactive Strength Index, ICC= Interclass correlation, 95% CI= 95% confidence interval, CV= Coefficient of variation, and Lo-Up LOA= Lower and Upper 95% limits of agreement, Effect Size = Trivial < Bold Text, *= P< 0.05.

Isometric Mid-Thigh Pull

Supplementary Table 4: Between trial test-retest reliability of IMTP performance

		Placebo				Caffeine			
		PL Trials	T1 vs T2	T1 vs T3	T2 vs T3	CF Trials	T1 vs T2	T1 vs T3	T2 vs T3
Peak Force (N. Kg)	ICC	.324	.323	.141	.652	.875	.863	.923	836
	95% CI	.062-.597	-.066-.639	-.283-.522	.334-.838	.766-.941	.697-941	.826-967	.648-928
	Significance	.007*	.053	.260	.001*	.001*	.001*	.001*	.001*
	CV	13.1%	13.1%	14.5%	8.2%	4.5%	4.5%	4.3%	5.1%
	Mean bias		2.81	1.96	-.85		-.91	-.47	.44
	Lo-Up LOA		-11.1-18.8.	-15.0-19.0	-8.4-6.7		-5.9-4.1	-4.0-3.1	-5.1-6
	ICC	.367	.520	.454	.077	.416	.193	.398	.684

Time to peak force (Sec)	95% CI	.104-.631	.128-.769	.061-.728	-.333-.470	.154-.668	-.253-.566	-.033-.700	.381-855.
	Significance	.003*	.006*	.014*	.361	.001*	.195	.034*	.001*
	CV	40%	34.9%	39.1%	46%	37%	39.8%	38.5%	32.5%
	Mean bias		-.12	.35	.47		-.19	.01	.19
	Lo-Up LOA		-3.14-2.91	-2.42-3.13	-3.07-4.02		-3.48-3.10	-2.94-2.94	-1.85-2.23
Force (N) at 100 m/s	ICC	.740	.748	.674	.817	.614	.668	.635	.548
	95% CI	.552-872	.481-.887	.356-.851	.608-.920	.381-.799	.357-.847	.301-.830	.165-.785
	Significance	.001*	.001*	.001*	.001*	.001*	.001*	.001*	.004*
	CV	11%	9.3%	12.7%	10.2%	12.4%	10.6%	9.3%	14.6%
	Mean bias		-1	3	4		-57	-47	10
	Lo-Up LOA		-461-458	-552-557	-437-445		-652-538	-717-623	-647-667
Force (N) at 300 m/s	ICC	.746	.750	.800	.699	.745	.245	.761	.337
	95% CI	.561-.875	.489-.888	.576-.912	.404-.863	.559-.874	-.204-.602	.510-.893	-.089-.659
	Significance	.001*	.001*	.001*	.001*	.001*	.138	.001*	.060
	CV	9.2%	8%	9.5%	10%	8.3%	7.3%	8.8%	8.8%
	Mean bias		41	-19	-60		43	-48	-91
	Lo-Up LOA		-562-643	-680-641	-825-705		-1120-1205	-647-551	-1097-915

Note: T1= Trial 1, T2= Trial 2, T3= Trial 3, ICC= Interclass correlation, 95% CI= 95% confidence interval, CV= Coefficient of variation, and Lo-Up LOA= Lower and Upper 95% limits of agreement, Effect Size = Trivial < Bold Text, *= P< 0.05.

Effect of Caffeine on Repetition until Failure

Supplementary Table 5: Between trial test-retest reliability of repetitions until failure during Chest Press, Shoulder Press, Squats, and Deadlifts.

		Placebo				Caffeine			
		PL Trials	T1 vs T2	T1 vs T3	T2 vs T3	CF Trials	T1 vs T2	T1 vs T3	T2 vs T3
CP Set 1	ICC	.723	.514	.489	.761	.656	.605	.685	.793
	95% CI	.527-.862	.118-.767	.106-.749	.508-.893	.435-.824	.248-.816	.379-.856	.556-.909
	Significance	.001*	.007*	.008*	.001*	.001*	.001*	.001*	.001*
	CV	17.1%	17.6%	16.6%	16.9%	13.2%	14.8%	14.2%	10.5%
	Mean bias		.14	-1.14	-1.27		.23	.50	.27
	Lo-Up LOA		-7 - 8	-10 -8	-8 - 5		-7 - 8	-6 - 7	-5 - 6
CP Set 2	ICC	.525	.487	.481	.731	.512	.636	.620	.792

	95% CI	.273-.742	.083-.751	.098-.743	.452-.879	.258-.734	.311-.830	.283-.822	.561-.908
	Significance	.001*	.011*	.009*	.001*	.001*	.001*	.001*	.001*
	CV	18.9%	19.4%	20.1%	17.2%	14.5%	13.4%	17%	13%
	Mean bias		-.27	-1.00	-.73		.73	.73	.00
	Lo-Up LOA		-8 - 8	-8 – 6	-6 - 5		-5 - 7	-6 - 8	-5 -5
SP Set 1	ICC	.476	.525	.432	.202	.694	.577	.564	.660
	95% CI	.217-.709	.079-.783	.010-.720	-145.-.539	.486-.846	.227-.797	.191-.793	.346-.842
	Significance	.001*	.001*	.023*	.132	.001*	.002*	.003*	.001*
	CV	18.2%	18.2%	13.6%	22.9%	13.2%	15.6%	13.2%	10.6%
	Mean bias		1.91	-.09	-2.00		.95	.32	-.64
	Lo-Up LOA		-3 - 7	-6 - 5	-9 – 5		-6 - 8	-6 - 7	-6 – 5
SP Set 2	ICC	.453	.321	.258	.467	.571	.297	.329	.402
	95% CI	.192-.693	-.120-.652	-.178-.608	.080-.736	.326-.772	-.115-.628	-098.-.653	-018.-.700
	Significance	.001*	.073	.121	.011*	.001*	.080	.065	.031*
	CV	20.2%	21.8%	17.9%	21.1%	15.7%	16.3%	15.9%	14.8%
	Mean bias		.27	-.55	-.82		1.00	.55	-.45
	Lo-Up LOA		-7 - 7	-7 - 6	-7 - 5		-6 - 8	-5 - 7	-7 – 6
SQ Set 1	ICC	.551	.746	.491	.376	.349	.487	.581	.659
	95% CI	.303-.760	.484-.886	.101-.751	-027.-.679	.086-.617	.086-.751	.218-.802	.332-.844
	Significance	.001*	.001*	.009*	.036*	.004*	.010*	.002*	.001*
	CV	14.5%	11.2%	14.1%	18.3%	15.1%	18.2	14.9%	12.3%
	Mean bias		.50	-.73	-1.23		-.50	-.55	-.05
	Lo-Up LOA		-5 - 6	-8 - 7	-10 - 7		-9 - 8	-9 - 8	-8 - 8
SQ Set 2	ICC	.507	.592	.430	.299	.384	.564	.622	.840
	95% CI	.252-.731	.248-.806	.010-.718	-.137-.636	.121-.644	.199-.792	.275-.825	.653-.930
	Significance	.001*	.001*	.023*	.087	.002*	.003*	.001*	.001*
	CV	16.6%	12.4%	17.8%	19.6%	12.1%	13.6%	14%	8.7%
	Mean bias		.91	.27	-.64		-.59	.14	.73
	Lo-Up LOA		-6 – 7	-7 - 8	-9 - 8		-7 - 6	-6 - 7	-3 - 5
DL Set 1	ICC	.741	.709	.761	.701	.682	.636	.773	.668
	95% CI	.554-.872	.426-.876	.505-.894	.413-.863	.470-.839	.304-.830	.525-.899	.356-.847

	Significance	.001*	.001*	.001*	.001*	.001*	.001*	.001*	.001*
	CV	15.5%	17.8%	12%	16.6%	15%	17.9%	17.2%	9.9%
	Mean bias		.91	-.18	-1.09		.68	-.14	-.82
	Lo-Up LOA		-5 - 7	-7 - 7	-8 - 6		-8 – 9	-9 - 8	-10 - 8
DL Set 2	ICC	.703	.753	.773	.705	.610	.601	.409	.758
	95% CI	.498-.851	.496-.889	.526-.899	.415-.886	.375-.796	.244-.813	.019-.706	.506-.891
	Significance	.001*	.001*	.001*	.001*	.001*	.001*	.030*	.001*
	CV	18.4%	22.2%	12.4%	20.8%	18.1%	19.2%	23.7%	11.5%
	Mean bias		1.00	-.05	-1.05		.27	-.18	-.45
	Lo-Up LOA		-5 - 7	-5 - 5	-6 - 4		-7 - 7	-8 - 8	-5 – 4

Note: RTF= Reps until Failure, CP= Chest Press, SP= Shoulder Press, SQ= Squats, DL= Deadlifts, T1= Trial 1, T2= Trial 2, T3= Trial 3, ICC= Interclass correlation, 95% CI= 95% confidence interval, CV= Coefficient of variation, and Lo-Up LOA= Lower and Upper 95% limits of agreement, Effect Size = Trivial < Bold Text, * = P< 0.05.

Effect of Caffeine on Rate of Perceived Exertion

Supplementary Table 6: Between trial test-retest reliability of rate of perceived exertion following repetitions until failure protocol.

		Placebo				Caffeine			
		PL Trials	T1 vs T2	T1 vs T3	T2 vs T3	CF Trials	T1 vs T2	T1 vs T3	T2 vs T3
CP Set 1	ICC	.428	.234	.450	.452	.637	.728	.504	.602
	95% CI	.166-.676	-.217-.595	.035-.729	.042-.730	.409-.813	.448-.878	.104-.761	.264-.811
	Significance	.001*	.150	.018*	.017*	.001*	.001*	.008*	.001*
	CV	4.4%	5.3%	3.4%	4.6%	3.3%	2.2%	4.0%	3.7%
	Mean bias		-.09	.09	.18		-.36	.00	.36
	Lo-Up LOA		-4-4	-2-2	-3-3		-2-1	-3-3	-2-3
CP Set 2	ICC	.448	.619	.511	.394	.466	.327	.426	.406
	95% CI	.187-.690	.288-.820	.140-.760	-.037-.697	.206-.703	-.070-.644	.045-.707	-.020-.704
	Significance	.001*	.001*	.005*	.036*	. 001*	.055	.012*	.031*
	CV	2.9%	2.2%	3%	3.5%	3.3%	3.4%	3.7%	2.8%
	Mean bias		.36	.32	-.05		-.41	-.50	-.09
	Lo-Up LOA		-2-2	-2-2	-3-3		-3-2	-3-2	-2-2
SP Set 1	ICC	.398	.457	.574	.319	.326	.210	.173	.195

	95% CI	.135-.654	.080-.727	.210-.798	-.095-.643	.064-.599	-.242-.579	-.270-.550	-.252-.567
	Significance	.001*	.009*	.002*	.066	.007*	.177	.220	.193
	CV	3.7%	3.8%	2.8%	4.4%	5.6%	6.1%	6.0%	4.6%
	Mean bias		-.50	-.14	.36		-.14	-.32	-.18
	Lo-Up LOA		-3-2	-2-2	-2-3		-5-5	-5-4	-3-3
SP Set 2	ICC	.163	-.098	.249	.149	.495	.359	.649	.269
	95% CI	.080-.459	-.502-.333	-.201-.605	-.298-.535	.238-.722	-.076-.676	.317-.838	-.174-.617
	Significance	.102	.670	.134	.255	.001*	.051	.001*	.113
	CV	4.8%	4.6%	4.7%	5.1%	2.9%	3.2%	2.6%	3%
	Mean bias		-.27	-.09	.18		.09	-.05	-.14
	Lo-Up LOA		-3-3	-4-3	-3-4		-2-2	-2-2	-2-2
SQ Set 1	ICC	.505	.501	.427	.382	.347	.150	.482	.482
	95% CI	.250-.729	.130-.754	.029-.712	-.009-.681	.084-.615	-.231-.514	.081-.748	.081-.748
	Significance	.001*	.004*	.020*	.014*	.004*	.228	.011*	.011*
	CV	4.1%	3.8%	3.6%	4.5%	4.1%	4.4%	4.6%	3.1%
	Mean bias		-.50	.32	.82		-.59	-.73	-.14
	Lo-Up LOA		-3-2	-2-3	-2-3		-4-2	-3-2	-2-2
SQ Set 2	ICC	.556	.339	.727	.451	.409	.181	.520	.308
	95% CI	.309-.763	-.041-.648	.448-.877	.071-.723	.146-.662	-.226-.545	.121-.771	-.121-.640
	Significance	.001*	.038*	.000*	.011*	.001*	.196	.002*	.078
	CV	2.8%	3.3%	2.1%	3%	3.2%	3.2%	3.2%	3.1%
	Mean bias		-.50	-.09	.41		-.36	-.55	-.18
	Lo-Up LOA		-3-2	-2-2	-2-3		-3-2	-2-1	-2-2
DL Set 1	ICC	.212	.077	-.030	.272	.642	.553	.601	.619
	95% CI	.038-.504	-.373-.484	-.411-.376	-.122-.606	.416-.815	.181-.786	.262-.810	.281-.821
	Significance	.051	.370	.557	.091	.001*	.003*	.001*	.001*
	CV	7.6%	9.7%	6.3%	6.7%	2.6%	3%	2.3%	2.6%
	Mean bias		-.09	-.73	-.82		-.18	-.36	-.18
	Lo-Up LOA		-6-6	-5-4	-5-4		-2-2	-2-2	-2-2
DL Set 2	ICC	.165	.030	-.050	.264	.473	.460	.400	.164
	95% CI	.078-.461	-.415-.447	-.440-.364	-.125-.598	.214-.708	.065-.733	-.016-.698	-290.-.547
	Significance	.098	.449	.593	.095	.001*	.014*	.031*	.236

CV	7.4%	9.8%	5.5%	6.9%	3.4%	3.4%	3.2%	3.8%
Mean bias		.14	-.77	-.91		.23	.18	-.05
Lo-Up LOA		-7-7	-6-5	-6-4		-2-2	-2.-2	-3-3

Note: RPE= Rate of Perceived Exertion, CP= Chest Press, SP= Shoulder Press, SQ= Squats, DL= Deadlifts, T1= Trial 1, T2= Trial 2, T3= Trial 3, ICC= Interclass correlation, 95% CI= 95% confidence interval, CV= Coefficient of variation, and Lo-Up LOA= Lower and Upper 95% limits of agreement, Effect Size = Trivial < Bold Text, *= $P < 0.05$.
